# Molecular analysis of hepatitis B virus "a" determinant in asymptomatic and symptomatic Mexican carriers

**DOI:** 10.1186/1743-422X-4-6

**Published:** 2007-01-11

**Authors:** Martha-Eugenia Ruiz-Tachiquín, Hilda-Alicia Valdez-Salazar, Vicencio Juárez-Barreto, Margarita Dehesa-Violante, Javier Torres, Onofre Muñoz-Hernández, Ma-Teresa Alvarez-Muñoz

**Affiliations:** 1 Unidad de Investigación en Enfermedades Infecciosas, Unidad Médica de Alta Especialidad (UMAE)-Pediatría, Centro Médico Nacional-Siglo XXI, Instituto Mexicano del Seguro Social, México DF, México; 2 Laboratorio de Análisis Clínicos, Unidad Médica de Alta Especialidad (UMAE)-Pediatría, Centro Médico Nacional-Siglo XXI, Instituto Mexicano del Seguro Social, México DF, México; 3 Clínica de Hepatitis, Departamento de Gastroenterología, Unidad Médica de Alta Especialidad (UMAE)-Hospital General. Centro Médico Nacional-Siglo XXI, Instituto Mexicano del Seguro Social, México DF, México

## Abstract

**Background:**

Hepatitis B virus (HBV) is a small DNA-containing virus with 4 genes, *C*, *S*, *X *and *P*. The *S *gene codes for the surface antigen (HBsAg), which contains the "a" determinant, the main region for induction of a protective humoral immune response. To compare the genotype and sequence of the "a" determinant between strains isolated from asymptomatic and symptomatic Mexican HBV carriers.

**Results:**

21 asymptomatic (blood donors) and 12 symptomatic (with clinical signs and with >1 year lamivudine treatment) HBV carriers were studied; all patients were positive for the HBsAg in serum. Viral load, genotypes, and subtypes were determined in plasma. A fragment of the *S *gene including the "a" determinant was PCR amplified and sequenced to determine genotype, subtype and to identify mutations. Mean viral load was 0.7965 × 10^4 ^copies/ml in asymptomatic carriers and 2.73 × 10^6 ^copies/ml in symptomatic patients. Genotypes H, C, and F were identified in asymptomatic individuals; whereas H was dominant in symptomatic patients. A fragment of 279 bp containing the "a" determinant was amplified from all 33 carriers and sequences aligned with *S *gene sequences in the GenBank. Mutations identified were Y100N, T126I, Q129H and N146K in the asymptomatic group, and F93I and A128V in the symptomatic group.

**Conclusion:**

Differences in genotype and in mutations in the "a" determinant were found between strains from asymptomatic and symptomatic HBV Mexican carriers.

## Background

Hepatitis B virus (HBV) is a small, non-cytopathic virus with a circular partially double-strand DNA genome of approximately 3.2 Kb [1]. The genome has 4 overlapping genes: *PreS/S*, *PreC/C*, *X*, and *P*. The *Pre S/S *gene encodes for the three envelope proteins, large, middle, and small or HBsAg. The *PreC/C *gene encodes for the nucleocapsid protein and HBeAg. The *X *gene encodes for the transactivating protein X and the *P *gene encodes for polymerase with reverse transcriptase (RT) and RNase H activity [1]. The HBsAg contains the major epitopes for induction of a protective humoral immune response [2-6]. These epitopes are localized in the region known as the "a" determinant, between amino acid residues 99 and 169 [7-10], and are involved in the binding of antibodies against HBsAg. Amino acids changes in this region render mutant strains able to escape immune responses induced by vaccines [5,6,11,12]. Immune-escape mutants occur naturally [13-15], and in strains from patients with a weak or negative HBsAg reactivity in detection assays [10,15].

HBV strains are classified into 8 main genomic groups or genotypes, designated A through H [16-18], arbitrarily defined by an intergroup divergence of more than 8% based on the complete genomes [16,19-21]. The relationship between subtypes and genotypes has been reported, and several studies have described the geographic distribution for the different HBV subtypes/genotypes [16,17,20,22-25]. The study of HBV genotype has recently been focused on the clinical outcome of the infection, since studies suggest a possible association with the natural history and severity of the infection [26].

In Mexico, frequency of HBV chronic carriers is low, and the prevalence of HBsAg in blood donors ranges from 0.15 to 1.4% [27,28]; the frequency of hepatocellular carcinoma is also low [29]. Scarce studies have addressed genetic diversity and amino acids changes in HBsAg of HBV strains from Mexico [30]. The aim of the present work was to study genetic diversity in the *S *gene of HBV strains from asymptomatic and symptomatic carriers in Mexico. Genotype and mutations in the "a" determinant of the *S *gene leading to amino acids changes were compared between these two groups.

## Results

We studied 21 asymptomatic HBsAg carriers, 3 women (mean age 30 years old) and 18 men (mean age 42 years old), with average viral load of 0.7965 × 10^4 ^copies/ml; and 12 symptomatic patients, 5 women (mean age 50 years old) and 7 men (mean age 39 years old), with average viral load of 2.7 × 10^6 ^copies/mL (Table 1). Functional hepatic tests confirmed hepatic damage in symptomatic but not in asymptomatic cases (Table 1). Genotypes were determined by reverse hybridization and sequencing. Genotypes determined by reverse hybridization were H, C, F, C/H, C/F/H and indeterminate (ID) in asymptomatic cases, whereas in symptomatic patients only H and C/H genotypes were found (Table 2). On the other hand, the genotypes determined with the sequencing technique were only H, and C; genotype H was the predominant type (Table 2). In four asymptomatic cases the genotype could not be determined (ID) with the reverse hybridization technique, but was identified as genotype H with the sequencing method. Sequences of the "a" determinant were aligned with sequences of genotypes A-H (Z72478, X98077, AB048704, AB048701, AB032431, AB036907, AF160501, U91827, NC_003977) and analyzed to identify mutations. Mutations identified in HBV strains from the asymptomatic group were Y100N, T126I, Q129H and N146K; whereas in strains from the symptomatic group identified mutations were F93I and A128V. Subtypes found in the asymptomatic group were adw4q+, adw1q+, and adrq+; whereas in symptomatic patients subtypes detected where only adw4q+, and adrq+. Altogether, the genotype/subtype most frequent in our population was H/adw4q+ (Table 2).

**Table 1:** HBV viral load and hepatic function tests of asymptomatic and symptomatic carriers.

	Asymptomatic	Symptomatic
Test	Average ± standard deviation	Average ± standard deviation

HBV viral load (copies/mL)	7,965.57 ± 8,218.70	2,736,972.86 ± 4,553,767.67
Alanine aminotransferase (U/L)	26.0 ± 2.12	202.89 ± 19.80
Aspartate aminotransferase (U/L)	24.94 ± 8.84	104.26 ± 317.58
Direct bilirubin (mg/dL)	0.14 ± 0.03	0.26 ± 0.20
Total bilirubins (mg/dL)	0.53 ± 0.03	0.91 ± 0.25

**Table 2:** Genotypes and subtypes of the "a" determinant of HBV strains from asymptomatic and symptomatic cases, determined by both reverse hybridization and sequencing techniques.

			Genotyping of "a" determinant			
			
			Reverse hybridization		Sequencing	
Patient	Diagnosis	Viral load (copies/ml)	Bands stained	Genotype	Genotype/Subtype	Mutations

HBV1	Asymptomatic	477	11,15	H	H/adw4q+	Y100N
HBV2	Asymptomatic	3330	11,15	H	H/adw4q+	
HBV3	Asymptomatic	7350	11,15	H	H/adw4q+	Q129H
HBV5	Asymptomatic	32700	11,15	H	H/adw4q+	
HBV19	Asymptomatic	43100	15	ID*	H/adw4q+	
HBV20	Asymptomatic	5750	11,15	H	H/adw4q+	
HBV22	Asymptomatic	10300	8,9	C	H/adw4q+	
HBV24	Asymptomatic	1010	11,15	H	H/adw4q+	N146K
HBV25	Asymptomatic	4500	8,9,11,15	C/H	H/adw1q+	T126I
HBV26	Asymptomatic	8470	8,9,11,14,15	C/F/H	H/adw4q+	
HBV29	Asymptomatic	3650	11,15	H	H/adw4q+	
HBV30	Asymptomatic	869	11,15	H	H/adw4q+	
HBV32	Asymptomatic	2030	11,15	H	H/adw4q+	
HBV35	Asymptomatic	215	8,9	C	C/adrq+	
HBV37	Asymptomatic	19000	11	ID	H/adw4q+	
HBV38	Asymptomatic	3920	11,15	H	H/adw4q+	
HBV43	Asymptomatic	5990	14,15	F	H/adw4q+	
HBV48	Asymptomatic	20400	11,15	H	H/adw4q+	
HBV49	Asymptomatic	<200	none	ID	H/adrq+	
HBV50	Asymptomatic	428	none	ID	H/adw4q+	
HBV62	Asymptomatic	12100	11,15	H	H/adw4q+	
HBV7	Chronic hepatitis	682000	11,15	H	H/adw4q+	
HBV9	Chronic hepatitis	>200000	11,15	H	H/adw4q+	
HBV10	Chronic hepatitis	3970000	11,15	H	H/adw4q+	
HBV11	Chronic hepatitis	8070	11,15	H	H/adw4q+	
HBV12	Chronic hepatitis	<200	11,15	H	H/adw4q+	
HBV13	Chronic hepatitis	<200	11,15	H	H/adw4q+	
HBV14	Chronic hepatitis	<200	11,15	H	H/adw4q+	
HBV15	Chronic hepatitis	2560000	11,15	H	H/adw4q+	
HBV16	Chronic hepatitis	139000	11,15	H	H/adw4q+	F93I
HBV17	Chronic hepatitis	8480	11,15	H	H/adw4q+	
HBV18	Chronic hepatitis	3260	11,15	H	H/adw4q+	A128V
HBV21	Chronic hepatitis	6440000	8,9,11,15	C/H	C/adrq+	

Diversity in the amino acid sequence of the "a" determinant among strains from asymptomatic and symptomatic HBV Mexican carriers is presented in Table 3. T113S, S114T, R122K, I126T and P127L were the most frequent in both asymptomatic and symptomatic patients; whereas A159G, R160K, F161Y and V168A were more frequent in symptomatic patients.

**Table 3:** Frequency of aminoacid variants in the "a" determinant of HBsAg in strains from asymptomatic and symptomatic Mexican carriers.

amino acid variant	% in asymptomatic (n = 21)	% in symptomatic (n = 12)
T113S	79	92
S114T	84	92
R122K	95	100
I126T	95	92
P127V	5	0
P127L	79	92
A128V	0	8
P129L	5	0
Q129H	5	0
I150R	5	0
F158H	5	0
A159P	5	0
A159G	47	92
R160E	5	0
R160K	42	83
R160N	0	8
F161S	5	0
F161Y	42	83
L162Y	5	0
E164A	5	0
S167G	5	0
V168A	16	42
R169F	5	0

## Discussion

In this work we analyzed genetic diversity in HBV isolates from asymptomatic (blood donors) and symptomatic chronic carriers in Mexico. Genotypes were determined with a commercially available reverse-hybridization technique and compared with the sequencing technique. Reverse hybridization allows the detection of infection with mixed genotypes, suggesting that this technique is more reliable than sequencing to detect quasispecies present in lower proportion in the sample; although in some cases it failed to establish the genotype. On the other hand, besides genotyping, nucleic acid sequencing provides information on nucleotide and amino acid sequences of the region investigated, while other molecular methods detect only the point mutations specified by the probes or primers used.

Of interest, a higher diversity in the infecting genotypes was found among asymptomatic carriers (H, C, F, C/H, C/F/H), than in symptomatic patients, where genotype H was found in all cases and in only one case a C/H infection was detected. These results would suggest that in our population, genotype H strains are more prevalent than C and F genotypes in symptomatic cases. This observation is relevant since studies indicate certain association of the genotype with the clinical outcome of the infection; thus, in hepatocellular carcinoma, genotype C was more prevalent in patients >50 years and genotype B more prevalent in patients <50 years of age, than in age-matched asymptomatic carriers [37]. Of interest, in our study, genotype C was identified in four of the 21 asymptomatic cases. Alternatively, LMV treatment in the symptomatic cases might be selecting for the H genotype. Diversity of HBV genotypes may also affect the accuracy of diagnostic tests and therapeutic decisions [32].

HBV has been classified in eight genotypes, A through H [18], which show a geographic distribution. In this study, genotype H was found as the predominant genotype in Mexican strains, followed by genotypes C and F. Genotype H has a close phylogenetic relationship with genotype F [17,18]. The genotype C is reported as prevalent in Asian populations [20,33], and patients observed in our population might correspond to imported cases, due to the increased mobility among Asian and Latin-American countries; in fact, recent reports have documented the presence of HBV genotype H strains among Japanese blood donors [34], as an evidence of the global mobility. The genotype prevalent in a population may determine the type of mutations prevalent in the infecting strains [35]; for example, the immune-escape mutant G145R is closely associated with genotype D [36]. HBsAg has also been classified in subtypes based on the sequence of the *S *gene and in identification of the amino acids encoded at specific positions [33]; in our population, subtypes, like genotypes, were more diverse among strains from asymptomatic carriers than in symptomatic cases.

The hepatic function tests and HBV viral load showed that in our symptomatic patients there was hepatic damage and high viral replication, which suggests that the antiviral treatment with LMV was not working, even thought the drug was administered for at least one year in all cases. It is likely that after a long failed therapy, resistance to LMV has been developed. The main mutations associated with LMV resistance are located at the RT gene in the 204 position (rtM204I/V), which is in the catalytic YMDD motif. The *P *gene overlaps the *S *gene and thus mutations selected during antiviral treatment may cause concomitant changes to the overlapping reading frame; in particular altering the C-terminal region of HBsAg. Thus for mutations associated with LMV resistance, the rtM204V change is associated with a I195M change in the *S *gene (sI195M), while the rtM204I change is associated with three possible changes in the *S *gene, sW196S, sW196L, or a stop codon. In addition, mutation rtA181T is associated with the stop mutation sW172stop in the *S *gene [37]. However, in our study, none of these mutations were found in the *S *gene of the strains from symptomatic cases.

There has been several reports on HBV *S *gene mutants affecting amino acid position 120, 123, 124 126, 129, 131, 141, 144 and 145 of the "a" determinant and preS region. The most relevant mutations seem to be the substitutions of amino acid G145R, K141E and T131I and insertion of 3 amino acids between residues 123 and 124, since they markedly affect the antigenic structure of HBsAg [38]. Mexican strains of the present study showed mutations at the first and second loop of the "a" determinant; although it should be noted that these mutations differed between asymptomatic and symptomatic strains. Thus, in the asymptomatic cases mutations were found at positions 100, 126, 129, and 146, whereas in symptomatic patients mutation were in positions 93 and128; none of the above mutations are reported to affect significantly the antigenic structure of the protein. It should be emphasized that all cases included in the study were selected because they were HBsAg positive and so those patients with mutations rendering the S protein undetectable with the antibodies tested, were excluded.

## Conclusion

In conclusion, we report the genotypes of HVB prevalent in symptomatic and asymptomatic patients in Mexico and show that there is more genetic diversity and mutations in the "a" determinant of HBV strains infecting asymptomatic carriers than in symptomatic patients. In HBV strains from our population genotype H and subtype adw4q+ were predominant. The role of viral genotype and subtype, as well as the ethnicity of the host in the determination of the clinical outcome of HBV infection is still unclear, and studies on genotypes infecting populations should help to clarify this issue.

## Methods

### Serum and plasma samples

Blood samples were drawn from 21 asymptomatic blood donors, at the Central Blood Bank, Centro Médico Nacional-Siglo XXI (CMN-SXXI), Instituto Mexicano del Seguro Social (IMSS), and from 12 symptomatic patients attending the Hepatitis Clinic, Gastroenterology Service, General Hospital, CMN-SXXI-IMSS. All 33 patients were selected because they were known to be HBsAg carriers. Symptomatic cases were receiving lamivudine (LMV) treatment during at least one year. The study was approved by the IRB Committee of IMSS. Informed consent was obtained from all studied participants.

### Serology

Assay for HBsAg was performed in sera using a commercial HBsAg test (version 2 IMx, Abbott Laboratories, IL, USA) following manufacturer's instructions. Hepatic function tests (ALT, AST, Billirubins) were performed using the commercially available Dimension^® ^Clinical Chemistry System (Dade Behring, Inc. Neward, DE, USA).

### Viral load

HBV viral load was determined in plasma with a commercial quantitative assay (COBAS AMPLICOR HBV Monitor™, Roche Diagnostics, Indianapolis, IN, USA) according to manufacturer's instructions.

### DNA extraction

HBV DNA was extracted from plasma specimens with QIAamp^® ^Ultrasens^® ^Virus kit (QIAGEN) following manufacturer's instructions.

### PCR amplification of the S gene

A fragment of the *S *gene was amplified by nested PCR. Sequences of the primers and their relative positions were as follows: outer primers included HBV1-sense, 5'-CGC TGG ATG TGT CTG CGGCGT-3', position 371–391, and HBV2-antisense, 5'-CGA ACC ACT GAA CAA ATG GCA CT-3', position 682–704; inner primers were HBV3-sense, 5'-CAT CCT GCT GCT ATG CCT CAT CT-3', position 409–431 and HBV4-antisense, 5'-GGC ACT AGT AAA CTG AGC CA-3', position 668–687 (30). Ten μL of DNA extracted from plasma (template) were mixed with 40 μL of reaction mixture containing 1× PCR Buffer, 50 pmoles of each primer, 0.8 mM dNTPs, 2.5 mM MgCl_2_, and 2.5 U Taq polymerase (Amplificasa^® ^BIOGÉNICA, Mexico City, Mexico). Annealing and elongation for both primer pairs was for 90 sec at 47°C and 72°C, respectively. The first PCR was carried out for 45 cycles and the nested PCR for 40 cycles, using an initial denaturing step of 95°C for 2 min and a final amplification step of 72°C for 15 min  [39].

### Sequencing

PCR products (279 bp) were run in 2% agarose gel electrophoresis and the isolated band was extracted with a commercial kit (QIAquick^® ^Gel Extraction Kit, QIAGEN). The purified product was used for sequencing with the chain termination method, using Big Dye Terminator version 3.1 (ABI PRISM™, Foster City, CA, USA). Extension products were purified (DyeEx™ 2.0 Kit, QIAGEN) and separated in an automated DNA genetic analyzer (377 ABI PRISM™).

### Sequence analysis

Alignment of sequences was done using the Mega 3.1 program and analyzed manually by visual inspection.

### Genotyping

Genotyping was carried out in DNA extracted from plasma samples, using the INNO-LiPA HBV amplification and genotyping tests (Innogenetics, Ghent Belgium), following manufacturer's instructions.

### NCBI genotyping system

Genotype was also determined by sequence analysis of the 279 bp fragment from HBV "a" determinant, using the genotyping tool available at the National Library of Medicine's National Center for Biotechnology Information (NCBI) [40] using BLAST with a set of reference sequences with known genotypes [41].

## Competing interests

The authors declare that they have no competing interests.

## Authors' contributions

MERT: Designed and supervised most of the molecular studies; analyzed sequences and interpreted results. Participated in the preparation of the manuscript.

HAVS: carried out molecular techniques and participated in the analysis of results.

VJB: carried out immunoassays and interpreted the results.

MDV: responsible for the clinical aspects of the study, including the recruitment of patients; participated in the writing of the manuscript.

JT: Participated in the designed of the study and in the analyses and interpretation of the results. Critical review and edition of the manuscript.

OMH: Participated in the clinical designed of the study and in obtaining financial support for the study.

MTAM: responsible for the design of the study and for acquiring financial support. Supervision of the experimental work. Participated in the drafting of the manuscript.
